# Antiviral activity of salt-coated materials against SARS-CoV-2

**DOI:** 10.1099/acmi.0.000492.v5

**Published:** 2023-09-27

**Authors:** Christopher M. Coleman, Belinda Wang, Yixin Wang, Emmanuel Tapia-Brito, Ziwei Chen, James Riffat, Saffa Riffat, Rachael Tarlinton, Amir Ghaemmaghami

**Affiliations:** ^1^​ School of Life Sciences, University of Nottingham, Nottingham, UK; ^2^​ Wolfson Centre for Global Virus Research, University of Nottingham, Nottingham, UK; ^3^​ School of Veterinary Medicine and Science, University of Nottingham, Nottingham, UK; ^4^​ Department of Architecture and the Built Environment, University of Nottingham, Nottingham, UK

**Keywords:** coronaviruses, materials, respiratory medicine, virus stability

## Abstract

The SARS-CoV-2 pandemic demonstrated the importance of human coronaviruses and the need to develop materials to prevent the spread of emergent respiratory viruses. Coating of surfaces with antiviral materials is a major interest in controlling spread of viruses, especially in high-risk or high-traffic areas. A number of different coatings for surfaces have been proposed, each with their own advantages and disadvantages. Here we show that simple salt coating on a range of surfaces, including a novel biomass aerogel can reduce the infectivity of SARS-CoV-2 placed onto the surface. This suggests that a simple to apply coating could be applied to a range of materials and have an antiviral effect against SARS-CoV-2, as well as other potential emerging viruses.

## Data Summary

Supporting data including Supplementary Material 1 and 2 for Antiviral activity of salt-coated materials against SARS-CoV-2 are deposited at https://doi.org/10.6084/m9.figshare.22587607.v1 [1].

## Introduction

Human coronaviruses (hCoVs) are important human pathogens, but until recently have not caused significant disruption to society. hCoVs can be broadly grouped into seasonal and emerging hCoVs. The seasonal hCoVs, such as hCoV-299E and hCoV-OC43, usually cause mild ‘common-cold-like’ disease in healthy adults, but can occasionally cause significant outbreaks in settings with vulnerable populations, such as nursing homes (for example, [2]). Prior to 2020 there were two emerging hCoVs described: severe acute respiratory syndrome (SARS)-CoV-1 (previously known as SARS-CoV) and Middle East respiratory syndrome (MERS)-CoV. Both SARS-CoV-1 and MERS-CoV are zoonotic viruses that caused significant disease outbreaks, with high case fatality rates, but were (and, in the case of MERS-CoV, still are) geographically restricted. For MERS-CoV this is, in part, due to the distribution of the zoonotic source, the dromedary camel (*Camelus dromedarius*). The intermediate host for SARS-CoV-1 was the masked palm civet (*Paguma larvata*), which is not as geographically restricted, but was successfully eliminated from humans primarily through effective quarantine of infected individuals [3]. MERS-CoV continues to cause human infections, but is primarily a camel virus [4] and cannot spread between humans easily under normal conditions [5]. Therefore, neither SARS-CoV-1 nor MERS-CoV have reached pandemic level.

The current ongoing outbreak of SARS-CoV-2, however, has demonstrated the pandemic potential of coronaviruses emerging into the human population as hCoVs causing 694 million confirmed cases and 6.9 million deaths, as of August 2023, while spreading to nearly every country and continent in the world, including Antarctica (https://www.bbc.co.uk/news/world-europe-59848160).

Human coronaviruses have been recognized as a significant cause of common-cold-like illnesses since the 1960s, but despite the emergence of SARS-CoV-1, had not been seen as having major pandemic potential. Therefore, upon the emergence of SARS-CoV-2 we were not equipped with the tools needed to combat SARS-CoV-2. Despite significant advances in developing effective vaccines and new antiviral drugs against SARS-CoV-2, the constant emergence of new variants, waning immunity in vaccinated populations and drug side effects mean that personal protective equipment and biosecurity measures continue to play a major role in providing population-level protection against any new outbreaks. Hence, there is an urgent need to improve the efficacy of existing measures such as antiviral surfaces or face masks to prevent the spread of SARS-CoV-2 and future respiratory virus outbreaks.

The use of a variety of face coverings was one of the most widely adopted SARS-CoV-2 mitigation policies, despite considerable controversy as to the efficacy of specific policies [6, 7]. The properties of each mask, including material, fit to the face and filtration capacity can have a big impact on their efficacy [8, 9]. However, face masks coated with simple antiviral materials could be an important tool to prevent the spread of any virus. This is particularly the case when there is a novel virus, such as SARS-CoV-2, for which it will take some time to develop effective vaccines or drugs. An effective face mask may prevent the critical early spread of the virus and decrease viral load even if not eliminating exposure, effectively cutting off transmission at a time when the infection is still at a low enough level to be effectively managed and/or controlled.

Previous studies have coated materials with various coatings and many have shown antiviral effects (reviewed in [10]). But, salt coating of various materials has been proposed as an effective tool to prevent the spread of respiratory pathogens [11] and they have previously been shown to be antibacterial against a range of important human pathogens [12]. Additionally, salt coating of surfaces can be antiviral, both *in vitro* and, interestingly, *in vivo* [13, 14]. Specifically, coating surfaces prevented the spread of influenza viruses by inactivating viruses that passed through the coated filter [14] and reduced the stability of a pig coronavirus, transmissible gastroenteritis virus [13].

Here we describe a number of simple, cost effective and easily scalable materials that show antiviral activity against SARS-CoV-2 and could be rapidly deployed to prevent transmission in high-risk environments. The antiviral efficacy of coated surfaces was initially demonstrated using an animal orthobunyavirus namely Schmallenberg virus (SBV), which is not pathogenic in humans and can readily replicate in Vero E6 cells, the cells used for the SARS-CoV-2 work and, therefore, was a more readily reproducible surrogate for SARS-CoV-2.

Initial testing of antiviral efficacy of materials is typically done with a lower pathogenicity surrogate enveloped virus for safety reasons, in this case an animal orthobunyavirus, which is not pathogenic for humans, Schmallenberg virus (SBV) was used. A selection of coatings with high anti-SBV activity were then tested for their ability of neutralize SARS-CoV-2.

## Methods

### Materials

A full list of materials and coatings tested is provided in Supplementary Material 1 and 2.

**Fig. 1.**
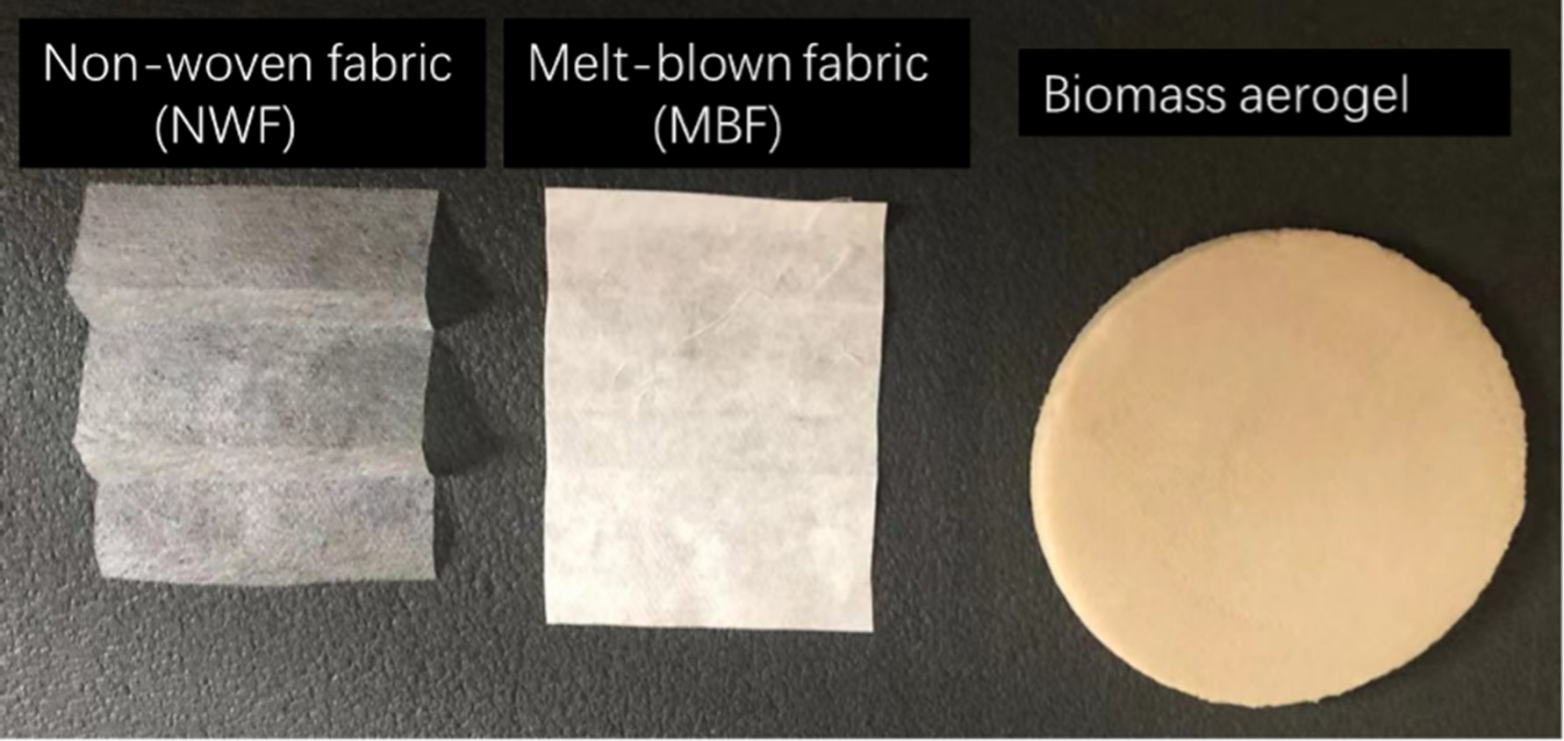
Single-layer images of the base materials used.

Table salt was purchased from Tesco PLC. Sodium chloride and potassium chloride were purchased from Fisher Scientific. Sodium percarbonate was purchased from Amazon. Biomass aerogels (Fig. 1) were developed and provided from the Hubei University of Technology [15]. Surgical mask was provided by KMD Company.

Non-sterile 12 cm by 10 cm pieces of non-woven fabrics and melt-blown fabrics (Fig. 1) were soaked in the 200 ml of each salt solution and then dried in a 50 °C drying oven. A small humidifier was used to spray 5 ml salt water onto the surface of non-sterile 15 cm^2^ pieces of biomass aerogels and then dried with a hairdryer. For the facemask (KMD Company), the inner and outer layers are non-woven fabric and the middle layer is melt-blown fabric. A similar biomass aerogel to the one used in this study has been previously reported [16, 17].

Surfaces were imaged using a scanning electron microscope (JEOL LV6060) and a lab-scale RS PRO USB digital microscope.

### Electron microscopy

The microstructure was observed with SEM (LV6060, JEOL, Tokyo, Japan). Before all the tests, samples were cut into 5 mm × 5 mm cubical pieces coated with gold particles using a gold and platinum sputter coater. Specimens were observed at different magnifications.

### Viruses and cells

Vero E6 cells were originally obtained from Professor Kin-Chow Chang (University of Nottingham) and maintained in minimal essential medium (MEM; Sigma) supplemented with 10 % foetal calf serum (FCS; Sigma), 1 % penicillin/streptomycin (Sigma) and 2 mM l-glutamine (Sigma).

The GLA-1 infectious variant of SARS-CoV-2 is an infectious clone developed from the original isolate of SARS-CoV-2 [18] and was obtained from the Centre for AIDS Reagents, NIBSC, UK. SARS-CoV-2 stocks were grown and quantified as described previously for other human coronaviruses [19].

Schmallenberg virus was obtained from the Frederich Loeffler Institute Germany was grown and quantified in Vero E6 cells as previously described [20] in Dulbecco’s Modified Eagle Media (DMEM; Sigma) supplemented with 10 % FCS (Sigma), and 2 mM l-glutamine (Sigma).

### Testing of antiviral activity of materials

In these assays, a 1-log drop in virus titre was considered an antiviral material. This is consistent with what would be required by the International Organisation for Standardisation (ISO) standards for antiviral activity of materials for both textile materials (ISO 21702) and non-porous surfaces (ISO 18184), though we did not attempt to meet all of those standards during these studies.

A non-sterile piece of each material (Supplementary Material 1) was excised from the main source material and placed into the well of a 96-well plate (Thermo Scientific) for SARS-CoV-2 or a 12-well plate (Thermo Scientific) for SBV. The material was cut to a size that comfortably fit flat onto the surface of the plate, but not so small that a 10 µl drop would not fit.

For Schmallenberg virus, 2.8×10^5^ TCID_50_ of infectious SBV was spotted onto the surface of the materials in 10 µl of fresh MEM supplemented with FCS (Sigma) and 2 mM l-glutamine (Sigma) for 15 min with the material. The material was then washed in 1 ml PBS to recover virus and then a 1 : 2 dilution of the wash was applied to Vero cells in 96 well plates for the TCID_50_ assay [20].

For SARS-CoV-2, 7.3×10^3^ TCID_50_ of infectious SARS-CoV-2 were spotted onto the surface in 10 µl of fresh Vero E6 growth media and left for 10, 30 or 60 min. After this, SARS-CoV-2 was washed from the surface in 200 µl of fresh MEM supplemented with FCS (Sigma), 2 mM l-glutamine (Sigma) and 1 % penicillin/streptomycin (Sigma). The amount of SARS-CoV-2 in the media was then quantified by TCID_50_ assay [19]. For RNA experiments, the same material samples with SARS-CoV-2, were submerged in 500 µl of TRIzol reagent (Ambion). The entire sample was recovered and the RNA was extracted using the DIrectZol kit (Zymo Research) according to the manufacturers’ instructions. SARS-CoV-2 RNA was quantified using primers targeted to the RNA-dependent-RNA polymerase [21]. SARS-CoV-2 RNA was assessed using the QuantiNova SYBR Green RT-PCR kit (Qiagen) and a FAST 7500 Real-Time PCR System (Applied Biosystems), both according to the manufacturers’ instructions. *C*_t_ values for ‘positive’ samples were in the range of 25–35 (data not shown). Negative samples often gave no *C*_t_ value, these were assigned the number 40 (the maximum possible cycle number) for calculation purposes (data not shown). Relative expression was determined using the delta*C*_t_ method, compared to the no material control (i.e. SARS-CoV-2 in the well of a 96-well plate).

### Quantification of Schmallenberg and SARS-CoV-2 viruses by TCID_50_ assay

Schmallenberg virus TCID_50_ was performed as previously described [20]. Schmallenberg virus in suspension in cell culture media was used as a positive control and the cell culture medium with no virus as a negative control. A % reduction in virus titre compared to the control and a log reduction in virus concentration was calculated.

SARS-CoV-2 TCID_50_ assay was performed using the same method as previously described for other coronaviruses [19]. Relative recovered SARS-CoV-2 was calculated by comparison to a no material control.

### Statistics

All data were analysed using a one-way ANOVA and Dunnetts’ multi-comparison test using Prism (GraphPad). Statistical significance was assumed where *P*<0.05.

## Results and discussion

**Fig. 2.**
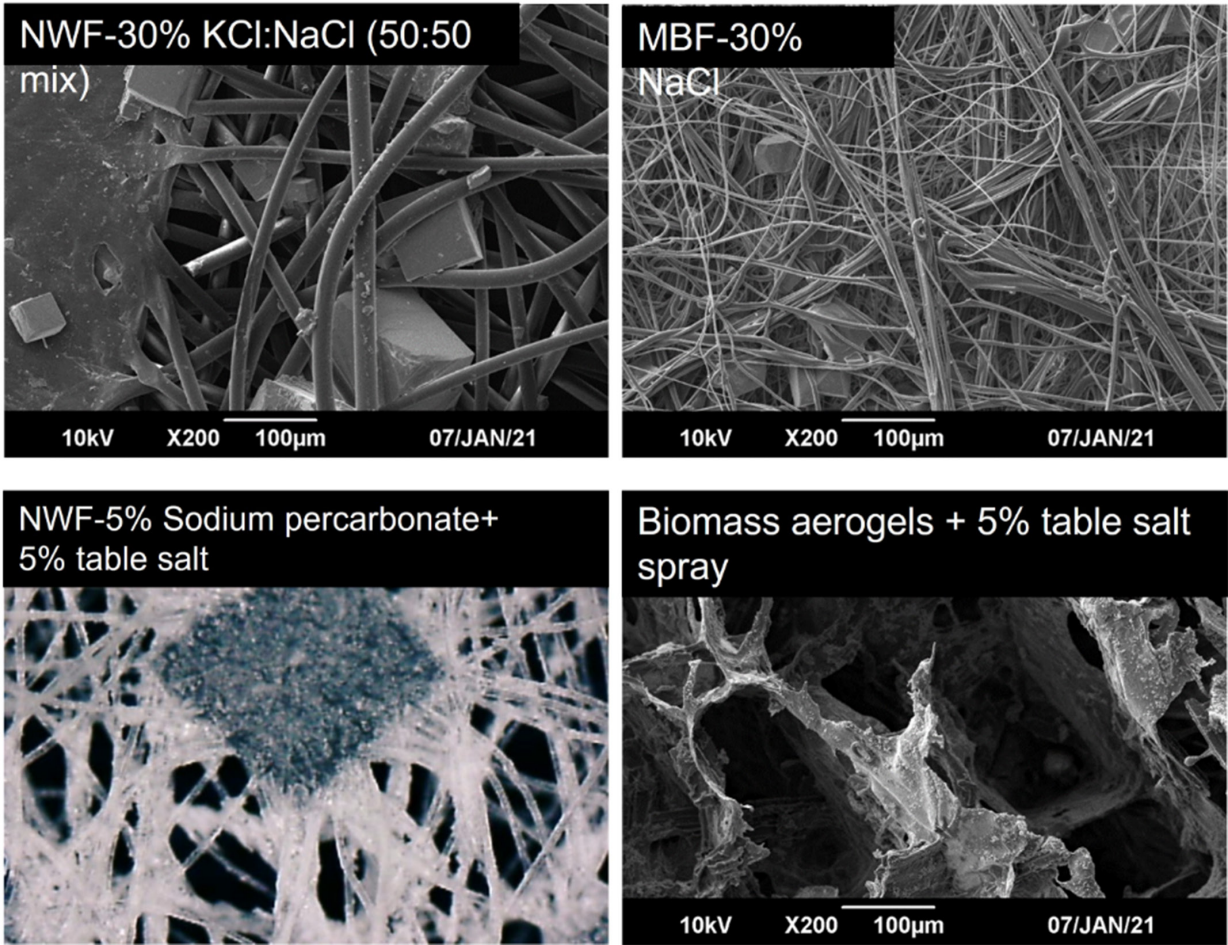
Salt deposit onto various surfaces.

Successful deposit of salt onto surfaces was confirmed through the observation of small salt particles in the materials using SEM (Fig. 2) and digital microscopy (data not shown).

### Preliminary screening of materials for antiviral activity against Schmallenberg virus

Initially all materials listed in Supplementary Material 1 were tested for antiviral activity against Schmallenberg virus. These results yielded 16 materials that showed antiviral activity against Schmallenberg virus (materials highlighted in grey in Supplementary Material 1). SBV was chosen for these experiments because it is easier to handle and readily replicates in Vero E6 cells, the cells used for the SARS-CoV-2 work and, therefore, was a more readily reproducible surrogate.

Schmallenberg virus was used as alternative commonly used surrogate viruses (such as feline coronavirus or low pathogenicity avian influenza) do not always grow readily in the same cell lines as SARS-CoV-2. Additionally, tools for other hCoVs (apart from SARS-CoV-1 and MERS-CoV, which have the same biosafety concerns as SARS-CoV-2) are often less well developed and the viruses do not always cause the robust cell death that allows for a rapid screening process.

### Antiviral activity of materials against SARS-CoV-2

The antiviral materials from the Schmallenberg screen (Supplementary Material 1, highlighted in grey) and some additional materials (Supplementary Material 2) were tested for antiviral activity against SARS-CoV-2. Virus was added to the surface and left in contact for 10 min and, then, recovered virus quantified by TCID_50_ assay. In line with ISO standards 21 702 and 18 184, A material was considered to be a ‘hit’ if the virus titre was lowered by at least 1-log from the control (SARS-CoV-2 on the surface of the 96-well plate) run in parallel. All ‘hit’s from the Schamallenberg virus screen also showed antiviral activity against SARS-CoV-2 and, all together these results yielded 16 materials that showed antiviral activity against SARS-CoV-2 virus (materials highlighted in grey in Supplementary Material 1 and Supplementary Material 2).

**Fig. 3.**
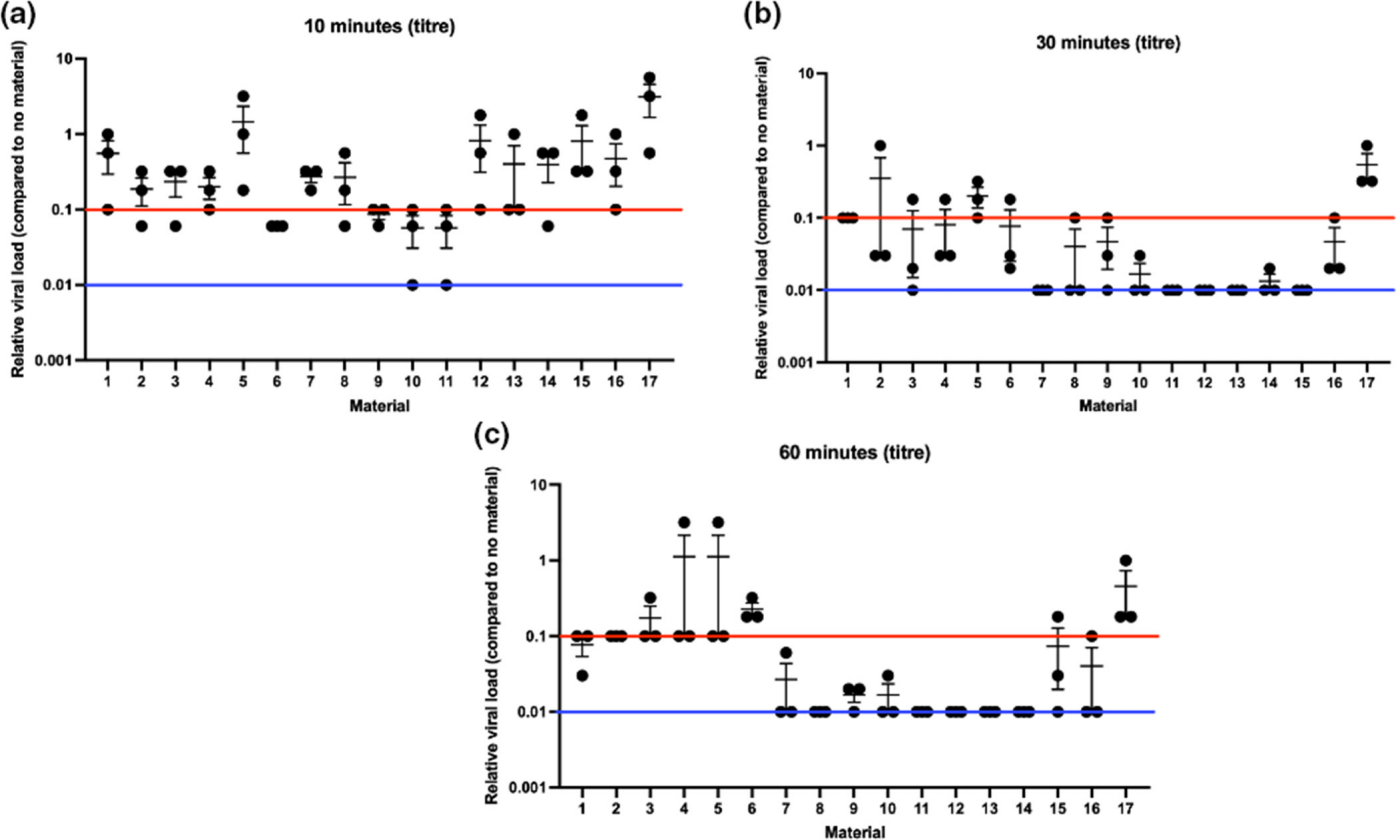
Recovered SARS-CoV-2 after 10 min (**a**), 30 min (**b**) or 1 h (**c**) exposure to materials. Numbers correspond to materials stated in Table 1. Red line indicates a 1-log drop compared to control. Blue line indicates limit of detection. Individual data points and the mean±sem are shown.

To further determine the anti-SARS-CoV-2 activity of each material, the data from the screen were repeated and antiviral activity was also tested over a longer contact time. For ease of labelling, each hit material and one control (a material that had no effect on virus titre) was assigned a number 1–17. The 17 materials in Table 1 were tested for anti-SARS-CoV-2 activity at 10, 30 and 60 min post-SARS-CoV-2 addition (Fig. 3).

**Table 1.** Numbers identifying each material in figures

Assigned no.	Material	
1	Face mask coated with 30 % NaCl:KCl (50 : 50 mix)	Middle layer (melt-blown fabric)
2	Face mask coated with 30 % NaCl	Middle layer (melt-blown fabric)
3	Non-woven fabric coated with sodium percarbonate at shown %	5 % + 5 % table salt
4		5 %+3 % table salt
5	Bioaerogel with 20 % salt and 2 % TiO_2_	
6	Table salt spray on non-woven fabric at shown %	5
7		10
8		15
9		20
10	30 % KCl:NaCl (50 : 50 mix) on non-woven fabric	
11	Bioaerogel-KIG2S4W52 at shown %	50+5 % salt spray
12		70+5 % salt spray
13		90+5 % salt spray
14		50+20 % salt spray
15		70+20 % salt spray
16		90+20 % salt spray
17 (control)	Uncoated face mask material	Middle layer (melt-blown fabric)

At 10 min post-SARS-CoV-2 addition (Fig. 3a), the results were variable, with some materials showing more variation than others. However, materials 6, 9, 10 and 11 showed consistent recovered titres of greater than 1-log reduction compared to the no coating control (Fig. 3a, red line shows 1-log drop). None of the coatings at this time point consistently achieved no virus recovery (Fig. 3a, blue line shows detection limit). When analysed statistically all samples, except sample 5, showed statistically significantly different titres compared to the control (one way ANOVA and Dunnetts’ multi-comparison test; *P*<0.05), suggesting that the coatings did significantly affect SARS-CoV-2 stability.

By 30 min post-SARS-CoV-2 addition (Fig. 3b), materials 1, 7, 8, 9, 10, 11, 12, 13 14, 15 and 16 were able to consistently lower the virus titre by at least 1-log compared to the no coating control. There was no SARS-CoV-2 recovered from materials 7, 11, 12, 13 or 15 (Fig. 3c). Only material 5, did not consistently show at least a 1-log drop in recovered SARS-CoV-2 titre compared to the no coating control (Fig. 3b). When analysed statistically all samples, except samples 2 and 5, showed statistically significantly different titres compared to the control surface (one way ANOVA and Dunnetts’ multi-comparison test; *P*<0.05), suggesting that the coatings did significantly affect SARS-CoV-2 stability.

By 60 min post-SARS-CoV-2 addition (Fig. 3c), all materials, including material 5, were able to consistently lower the virus titre by at least 1-log compared to the no material control. There was no SARS-CoV-2 recovered from any material 8, 11, 12, 13 or 14 samples (Fig. 3c) and there was no recovered SARS-CoV-2 in 2/3 samples with materials 7 and 10 (Fig. 3c). Material 6, did not consistently show at least a 1-log drop in recovered SARS-CoV-2 titre compared to the no material control (Fig. 3c), but did show a drop in titre compared to control of approximately 67 %. When analysed statistically none of the samples were significantly different from the control surface (one way ANOVA and Dunnetts’ multi-comparison test; *P*>0.05), suggesting that the coatings did not significantly affect virus stability. However the uncoated surface also had a significant drop in titre compared to the no material control, so exposure to any surface at this time point reduced SARS-CoV-2 stability.

Overall, all of the tested materials were able to significantly drop SARS-CoV-2 titre over time, with some showing complete destruction of SARS-CoV-2 infectivity.

### Detection of SARS-CoV-2 RNA after contact with surfaces

**Fig. 4.**
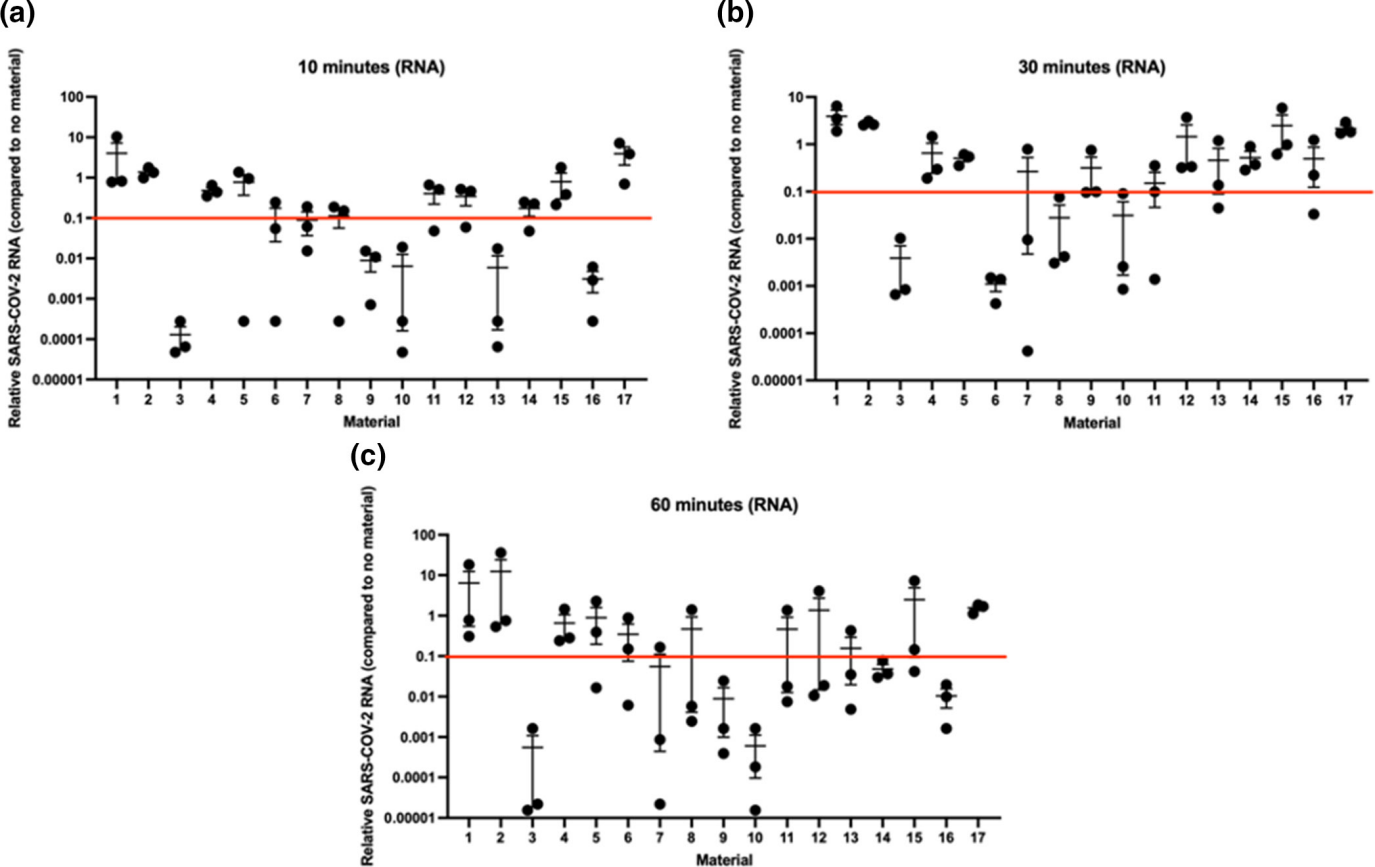
Recovered SARS-CoV-2 RNA after 10 min (**a**), 30 min (**b**) or 1 h (**c**) exposure to materials. Numbers correspond to materials stated in Table 1. Red line indicates a 1-log drop compared to control. Individual data points and the mean±sem are shown.

Because the only viral RNA that should be present in the virus preparation should be genomic RNA, this is a measure of physical virus presence in a sample. However, the virus may itself be either infectious or inactivated. To determine if there is evidence of SARS-CoV-2 RNA degradation, RT-PCR was performed on material samples that had had contact with SARS-CoV-2 for 10, (Fig. 4a), 30 (Fig. 4b) or 60 (Fig. 4c) minutes. These data were much more variable than the titre data, in that most samples did not show consistent reduction of SARS-CoV-2 RNA within or across timepoints (Fig. 4a–c; red line indicates a 1-log drop). Materials 3, 6, 8, 9, 10, 13, 14 and 16 consistently showed a 1-log or greater drop in SARS-CoV-2 RNA at at least one timepoint post-contact, but most of these did not show a drop across all timepoints. The other materials never consistently showed a greater than 1-log drop in SARS-CoV-2 RNA at any timepoint post-contact (Fig. 4a–c; red line indicates a 1-log drop). When compared statistically, none of the samples showed any significantly different RNA compared to the control surface (one way ANOVA and Dunnetts’ multi-comparison test; *P*<0.05 in all cases).

Although these data are highly variable, perhaps due to differences in the absorbance of the surfaces, we can at least conclude that SARS-CoV-2 RNA can still be detected in association with most of the surfaces at the various time points. Taken together with the TCID_50_ data, the data suggest this is not infectious SARS-CoV-2 suggests that fragments of SARS-CoV-2 RNA can remain on the surfaces for longer periods of time.

## Conclusions

Emerging new virus variants and waning immunity due to infection or vaccination mean that effective non-pharmaceutical intervention remains a critical part of protecting the public against ongoing and future outbreaks of SARS-CoV-2 and other respiratory viruses. In this study we identify a number of non-expensive and scalable salt formulations that, in line with the ISO standard a 1-log drop in titre, have antiviral activity against SARS-CoV-2 when used as a coating on common facemask fabrics and could, therefore, control spread of SARS-CoV-2.

Our observations are in line with previous studies that have shown salt coating of surfaces can be an effective tool to prevent the spread of respiratory pathogens [11] showing antibacterial [12] and antiviral [13, 14] properties. In this study we have shown a combination of Bioaerogel and salt spray are particularly effective in inactivating SARS-CoV-2 by at least 1-log after exposure of only 30 min, with 5 % salt spray showing this as early as 10 min post-exposure. Given that the presence of water has been specifically implicated to help with SARS-CoV-2 stability [22], it is reasonable to assume that the antiviral coatings is, at least partly, due to their potent desiccant properties. Interestingly, our data also indicate that the function of the antiviral coatings is not influenced by the nature of the substrate they are applied on. This means these salt coatings could be potentially applied on different existing materials and their use is not restricted to specific materials.

The detection of viral RNA on most of the surfaces suggest that that the surfaces do not cause the complete destruction of all viral components. Some of the materials, however, do appear to cause the complete degradation of SARS-CoV-2, such that not even fragments of SARS-CoV-2 RNA can be detected.

We acknowledge that these are *in vitro* studies that may not be applicable to the real-world, but these data are an important first step in the process. The use of the biomass aerogels, in particular, will be a key area of future study. We previously reported on the biophysical and filtration properties of similar biomass aerogels to those used in this study [16, 17]. One concern, for example, would be the breathability of novel facemask materials, such as the biomass aerogel [12]. Although we did not test this as part of this study, previous work suggests that a similar biomass aerogel had a low filtration resistance [16]. Additionally, although the pore size of biomass aerogels is large [17], we have previously showed that the overlapping network of pores creates a network that should block most viruses [17].

In short, in this study we have shown that spray coating of different types of fabric used in making facemasks provides potent antiviral properties against SARS-CoV2 and can be used as a fast and non-expensive method for developing more effective personal protective equipment against respiratory viruses. Further work will determine the exact mechanism of action of these coatings and determine the utility and efficacy of the antiviral masks in real-world settings.
